# Modelling aerosol-based exposure to SARS-CoV-2 by an agent based Monte Carlo method: Risk estimates in a shop and bar

**DOI:** 10.1371/journal.pone.0260237

**Published:** 2021-11-22

**Authors:** Henri Salmenjoki, Marko Korhonen, Antti Puisto, Ville Vuorinen, Mikko J. Alava

**Affiliations:** 1 Department of Applied Physics, Aalto University, Espoo, Finland; 2 Department of Mechanical Engineering, Aalto University, Espoo, Finland; Universite du Quebec a Montreal, CANADA

## Abstract

Present day risk assessment on the spreading of airborne viruses is often based on the classical Wells-Riley model assuming immediate mixing of the aerosol into the studied environment. Here, we improve on this approach and the underlying assumptions by modeling the space-time dependency of the aerosol concentration via a transport equation with a dynamic source term introduced by the infected individual(s). In the present agent-based methodology, we study the viral aerosol inhalation exposure risk in two scenarios including a low/high risk scenario of a “supermarket”/“bar”. The model takes into account typical behavioral patterns for determining the rules of motion for the agents. We solve a diffusion model for aerosol concentration in the prescribed environments in order to account for local exposure to aerosol inhalation. We assess the infection risk using the Wells-Riley model formula using a space-time dependent aerosol concentration. The results are compared against the classical Wells-Riley model. The results indicate features that explain individual cases of high risk with repeated sampling of a heterogeneous environment occupied by non-equilibrium concentration clouds. An example is the relative frequency of cases that might be called superspreading events depending on the model parameters. A simple interpretation is that averages of infection risk are often misleading. They also point out and explain the qualitative and quantitative difference between the two cases—shopping is typically safer for a single individual person.

## 1 Introduction

According to a recent study, the emerging new SARS-CoV-2 variants are extremely infectious with the basic reproduction number *R*_*o*_ ∼ 6 − 8. There is an urgent need to better understand the aerosol spread of such hyper-contagious respiratory viruses. In fact, the exposure of healthy people to infected individuals is a complex and multidisciplinary problem. Questions abound from how to provide scientifically reliable and preferably quantitatively meaningful and justified guidelines for the public and for the policies to restrain disease spreading to the desired target level to the fundamental statistics of spreading. The average reproduction number *R*_0_ and its variance are challenging to estimate a posteriori to say nothing about predictive, a priori modelling. In this work, we take a semi-quantitative approach to this problem by estimating exposure and risk from COVID-19 infected persons in certain relevant contexts. Following numerous earlier works [1–7] supported by growing body of experimental evidence [8–15], the airborne spread of SARS-CoV-2 via aerosolized virus is presently well established [16–18].

The main point of the present study is that the exposure and the resulting infection risk must be expressed in terms of probability distributions if one aspires for improved accuracy. From these, one of course is able to compute relevant quantities such as the average risk for exposure or contagion (with extra approximations). Here, we consider two interesting scenarios: a “super-market” and a “bar” [19–25]. The main difference in these two cases relates to the behavioral patterns of the “agents” or customers in both scenarios. This then becomes evident in the risk for such agents.

Our main objectives relate to the gaps left by the attempts to estimate quantitatively the statistics of risk. They are four-fold. First, from the practical viewpoint of understanding the risk involved with human activities clear quantitative differences are found: shopping is way less risky than going to a bar for typical person. Second, these risks can be tuned both by personal behavior (dwell time at the activity) and by an effort to decrease such risks (customer density, air ventilation to avoid any peaks in virus air concentration). Third, the concept of “super-spreaders” corresponds in our modelling to the tails of the distributions of the *number of persons infected / exposed by an infected person*. Finally, fourth, our simulation model and the results show that a combined approach involving physical detail (aerosols, geometry) and a consistent Monte Carlo modelling of a large cohort of individuals allows to obtain quantitatively relevant results. This indicates that a priori approaches in the same vein would often be of value for risk management.

This paper is organized as follows: Section 2 reviews the modelling details and the assumptions made for the choice of parameters. Section 3 looks at the two scenarios from the viewpoints of virus concentration fields, the statistics of exposure, and their coupling. It also presents alternative ways of assessing the results based on the Wells-Riley equation [26] and on the question if the contagion/exposure may be understood by simpler models. Conclusions, recommendations, and comparisons to other work are provided in Section 4.

## 2 Methods

### 2.1 The classical Wells-Riley model

A common model to estimate infection risks for respiratory diseases in confined spaces is the Wells-Riley model [11, 26, 27]. It assumes airborne transmission via infecting ‘quanta’ that are generated and emitted by infected agents in the space. Moreover, the quanta are assumed to be spatially uniformly distributed and inhaled by the healthy agents. The model then yields an estimate of probability of infection for the healthy given a time *t* spent in the indoor space with *I* infected people. The derived probability is [26]

$$\begin{array}{c} P_{infection}\left(t\right)=1-e^{-\frac{Iqpt}{Q}}, \end{array}$$ (1)

where *p* is the pulmonary ventilation rate, *Q* is the room ventilation rate and *q* is the infection quanta generation rate which is a disease dependent parameter.

### 2.2 Present Monte-Carlo model

**Fig 1:**
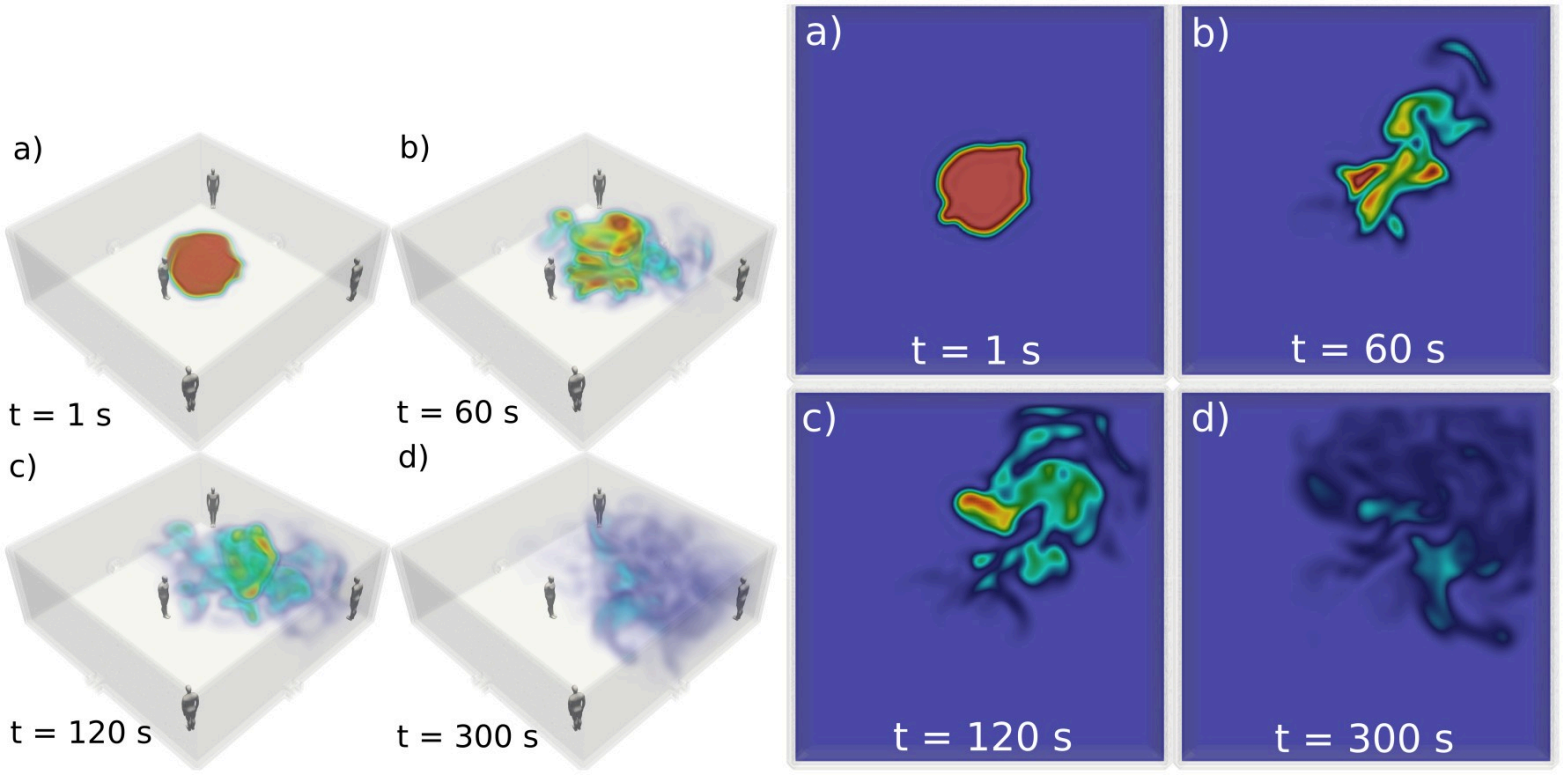
The figure on the left depicts the time-evolution of the concentration (arb. units) of an aerosol plume (red region) emitted by an infected person at the center of a room, where *t* = 0 s denotes the inception of the plume. The results displayed here were obtained in a CFD simulation incorporating the (turbulent) air currents generated by a typical room ventilation setup. The ventilation effectively dilutes, spreads and gradually removes the plume. The figure on the right illustrates this concentration data at the middle-cut plane with respect to the room height. The results in these figures indicate that in the event of an ideal ventilation, the plume can be considered to be subjected to isotropic turbulence. Therefore, the inception and mixing of the aerosol plume with the surrounding air can be modeled within a reasonable accuracy as a diffusion event with concentration sources and sinks.

Our model incorporates the spreading of aerosols from ‘infected’ agents [1, 28] and the measurement of exposure accumulated by the agents in the system. The concentration of aerosols *c*(*r*, *t*) follows the, diffusion equation

$$\begin{array}{c} \frac{\partial c}{\partial t}=D\Delta c+S-\frac{c}{\tau }, \end{array}$$ (2)

where we also have the source term *S* and sink term *c*/*τ*. The aerosol concentration applies to a reference volume of 1 m^3^. The approach adopted here is supported by Computation Fluid Dynamics (CFD) simulations performed in a room where the spreading of an aerosol plume is monitored over time while the air currents generated by the room ventilation are accounted for (see Fig 1). As seen in the figures, the ideal ventilation effectively subjects the plume to air currents undergoing isotropic turbulence, in which case the mixing of the plume with the surrounding air can be considered a diffusion event to a good accuracy. This lends credence to our simple and computationally inexpensive model for the aerosol concentration.

We simulate the concentration field in two dimensions and, thus, we assume that the diffusion occurs in the 2D grid made of cells with the size of the reference volume. The sink term then fixes the lifetime and decay of the airborne aerosol particles due to air ventilation. We run the simulations first with a value of *τ* = 100 s and then enlarge it to investigate the role of slower air circulation. The connection between parameter *τ* and “Air Changes per Hour” (ACH), the common measure of room ventilation, can be derived from Eq 2:

$$\begin{array}{c} \tau =\frac{1}{\text{ACH}}, \end{array}$$ (3)

so e.g. *τ* = 1200 s corresponds to ACH = 3.

Sources *S* of the field are point-like and come from the ‘infected’ agents in the system. They spread aerosols with both continuous emission of 5 aerosols / second, and discrete events [1, 29], or ‘coughs’, of e.g. *S*_0_ = 40000 aerosols with probability *p*_*c*_ = 6/3600 per second (i.e. on average 6 coughs per hour). The navigation of the agents in our model systems follows the same idea as the implementation in [1], where the agents behave as individuals interacting only to avoid collisions. As a slight improvement to the earlier model, the agents now avoid routes next to walls. This allows us to simulate more crowded systems without agents blocking each other and eventually jamming the entire system. The model describes rather well human dynamics modified by social distancing rules during the pandemic.

**Fig 2:**
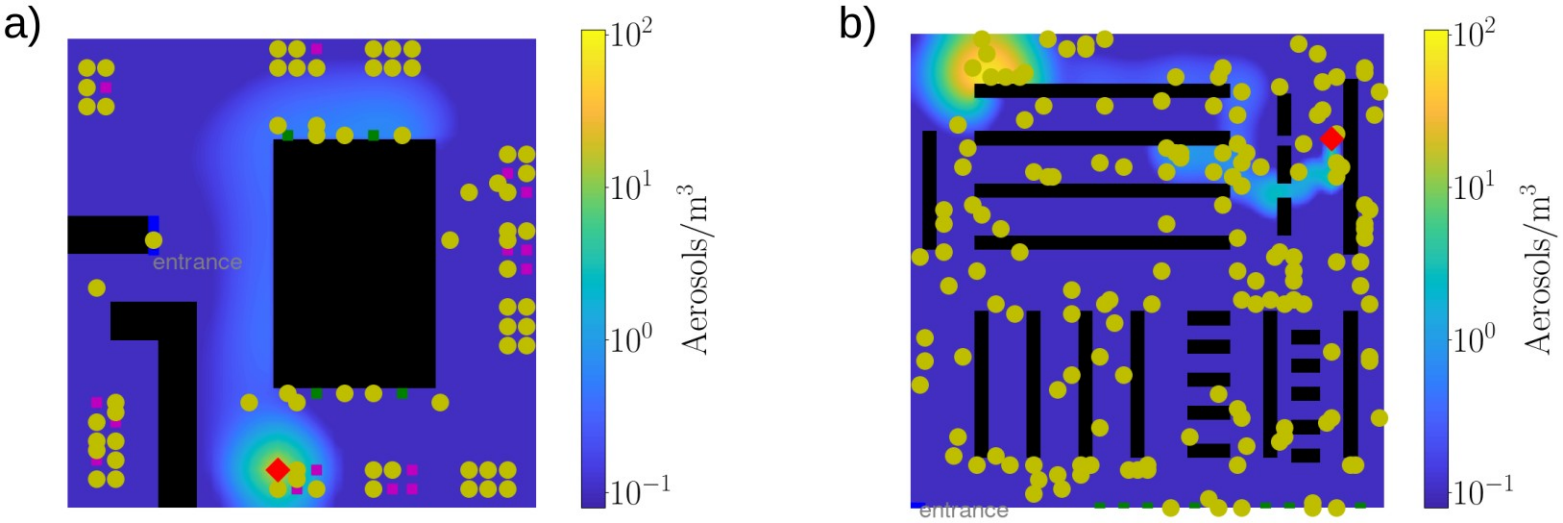
Snapshots from simulations in *(a)* bar and *(b)* supermarket environments. Healthy customers are depicted by yellow dots and infected customers by red diamonds, while the black regions are blocked, e.g. walls or shelves. In bar, the purple squares illustrate the seats available for the customers and the green squares are at the bar ‘counter’ where the agents get their next beverage. In supermarket, the green squares at the lower edge of the system are cashiers from where the customers leave the system. The colorbar shows the aerosol concentration in the systems. Opposed to the Wells-Riley model that assumes uniform distribution of infecting quanta, the aerosol concentrations in our model systems are non-uniform and highly dependent on the movement of the infected agent.

We implemented the simulations in two distinct environments. In ‘bar’ environment (with size *L*_*bar*_ = 50 × 50) as illustrated in Fig 2a, the customers enter the system and first navigate to one of the bartenders. After that, they find their designated, reserved seat where they spend a time drawn from Poisson distribution with mean of 16 minutes. The process of getting a drink from bar and enjoying it at the table is repeated 1-6 times with uniform distribution. The number of seats in the bar is restricted and while all seats are taken, no new customers enter the system.

For comparison, we replicated the simulations in a ‘supermarket’ (SM) environment (*L*_*SM*_ = 100 × 100) which is illustrated in Fig 2b. There the customers enter the system and are given a list of 1-60 positions (again, with uniform distribution) to visit before heading to the cashier and leaving the system. Therefore, the behaviour of the agent differs drastically between the two environments: in bar, the walkers are mostly stationary and in close contact with other customers. While in SM, they stay almost in constant movement.

The main parameter to measure during the simulations is the amount of inhaled aerosols *N* by our healthy agents. It is simply calculated as the accumulated aerosol concentration times the inhalation rate ${\dot{V}}_{inh}=0.33\;{\text{dm}}^{3}s^{-1}$, i.e.

$$\begin{array}{c} N={\dot{V}}_{inh}\int_{t_{0}}^{t_{1}}c\left(r,t\right)dt, \end{array}$$ (4)

where *t*_0_ and *t*_1_ are the times the agent enters and leaves the system, respectively, and *c*(*r*, *t*) are taken in the cells which the agent occupies. In addition to obtain an estimate on possible infections, we use the ‘critical’ limit of inhaled of aerosols *N*_*c*_ = 100 introduced in [1]. There it was estimated by comparing known cases of COVID-19 spreading events, that inhaling O(100) aerosols with a viral load could be a dose leading to transmission. However, we emphasize that this is only a rough estimate which gives us some way to compare our simulation results to previous models and real world. Another approach would be to use an estimate similar to the Wells-Riley model, and normalize the number of particles intaken by a person by a reference quantity. This could be then used to estimate for each agent a probability based on the stochastic history, based eg. on an exponentially growing likelihood, but the result would not be qualitatively different (essentially our choice amounts to state that for large enough aerosol loads the approaches are comparable).

To isolate the effect of single spreaders, we perform simulations where we first run the system to a steady-state (i.e. ∼ constant level of customers) which is dependent on the number of seats in bar or the influx of customers in SM. These values are chosen so that the eventual customer density *ρ* is comparable between the systems, as shown by Table 1. Once the system reaches the steady-state, we send in a single infected agent leading to an assumption of one spreader in the system instead of a proportion. The inhaled aerosols are then collected for the healthy customers that visit the system the same time or right after the infected customer. The simulations were repeated for 10000 times in bar and 500 times in supermarket.

**Table 1:** Average customer density in bars with varying number of seats and in supermarkets with varying customer influx. In SM, increase in *ρ* from larger influx is not linear as the system approaches jamming due to waiting times at cashiers.

Bar: Seats	*ρ*(customers/m^2^)	SM: Influx (customers / hour)	*ρ*(customers/m^2^)
32	0.013	360	0.009
48	0.020	720	0.018
64	0.027	1080	0.029
		1440	0.061

### 2.3 The Computational Fluid Dynamics (CFD) model

As indicated above, the primary results presented here are complemented by a full three-dimensional CFD simulation of an aerosol plume emitted by a person, exposed to air currents generated by the ventilation in a room. The approach is established on our previous work implementing a CFD solver labeled NS3dLab [30]. In this framework, the Navier-Stokes equations are solved on a continuum level utilizing the Chorin-Temam projection method while the aerosol plume is modeled by incorporating an additional transport equation for a passive scalar field normalized to the interval [0, 1]. The solver utilizes a pseudo-spectral approach in a periodic configuration, a skew-symmetric (kinetic energy conserving) form of the convection terms and a fourth order explicit Runge-Kutta time discretization. Further technical details are documented in [30].

The case presented in Fig 2 is initialized by setting the kinematic viscosity of the interstitial fluid to the value of air at NTP conditions. Additionally, two ventilation apertures are located near the floor level of the room (with cross-sectional dimensions of 0.3 m x 0.2 m). The inflow of air at these apertures is velocity controlled and a value of 1.0 m/s is used for a realistic ventilation setup. Additionally, a fine grid consisting of 224 x 224 x 88 nodal points is applied to model the space enclosed by the room (8 m x 8 m x 3 m), implying the simulation is performed close to the Direct numerical simulation (DNS) limit. Further, the passive scalar field is initialized to 1 in a spherical volume (radius 1 m) in front of the infectious person. This serves as a qualitative description of the behavior of an orally emitted aerosol plume in a closed indoor space with ventilation induced air flows.

### 2.4 ‘Zeroth order approximation’ for exposure

For simplification, we also derive a mean-field-like ‘zeroth order approximation’ for accumulated exposure by an average agent, *N*_0_. We start by assuming a static source of aerosols at the center of a 2D system with size *L* similar to our simulations. The source emits aerosols with a constant rate that is comparable to the average rate of our infected agents, i.e. 5 + *p*_*c*_ ⋅ *S*_0_ aerosols / second. Then, after the concentration field around the source reaches steady-state *c*(*r*, *t* → ∞), we add a customer that samples the field at a distance of two random points in a square, *r*_0_ ≈ 0.521*L*, for a time equal to the average spent in the system by the agents, i.e. 〈*t*_1_ − *t*_0_〉_*sim*_. Combining these we obtain,

$$\begin{array}{c} N_{0}={\dot{V}}_{inh}\cdot c\left(r_{0},t\to \infty \right)\cdot {\left\langle t_{1}-t_{0}\right\rangle }_{sim} \end{array}$$ (5)

for the zeroth order approximation. This is now system-dependent (bar/SM) as *r*_0_ and 〈*t*_1_ − *t*_0_〉_*sim*_ vary between the two simulation systems.

## 3 Results

### 3.1 Aerosol exposure

**Fig 3:**
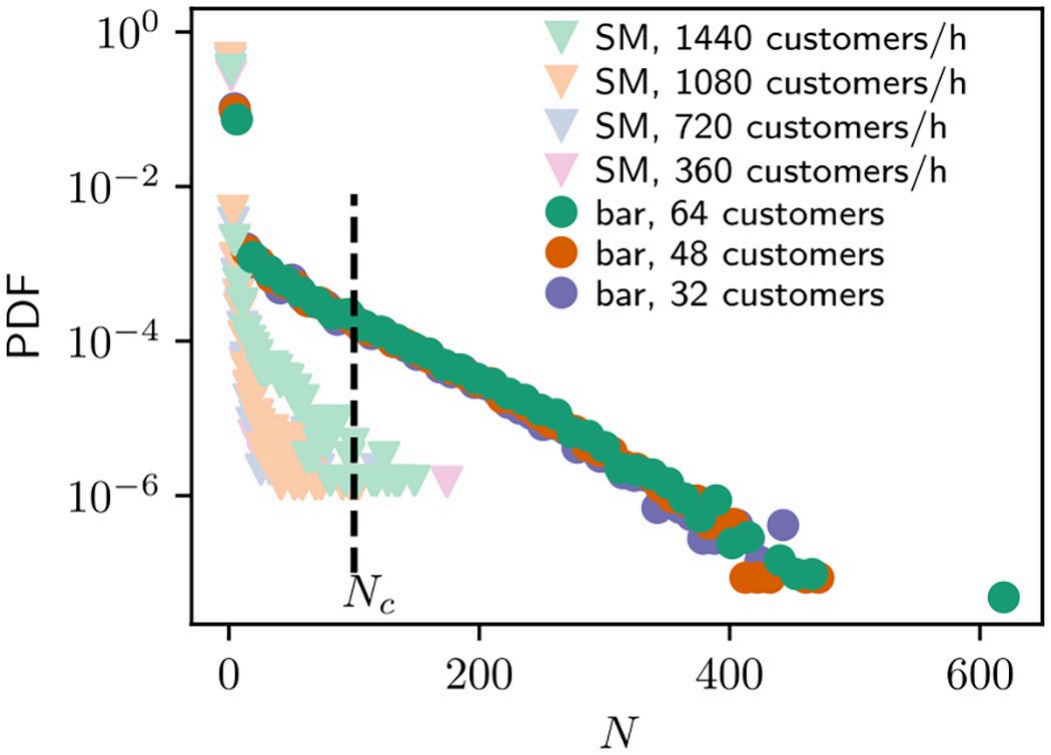
The distribution of accumulated exposure by healthy customers in supermarket simulations with varying customer density and in bar simulations with different number of seats.

The resulting distribution of inhaled aerosols by healthy customers in both environments is illustrated in Fig 3. The figure shows distinctly the two environments: in bar, the tail of the distribution is near exponential, and interestingly, with no clear difference caused by varying the amount of customers. In SM, the distributions vary with the customer density, as with less customers the distributions cease more sharply. More importantly one can focus on the levels of inhaled aerosols implied by the figure. Although clearly most customers survive with exposures close to zero aerosols in both bar and SM, there is a significant amount of bar customers with *N* > *N*_*c*_. Meanwhile SM simulations result in only few cases of critical spreading which agrees with the observations made in [1]. In the following section, we focus on the statistics of these critical spreading events.

### 3.2 Statistics of super-spreading and infection risk

**Fig 4:**
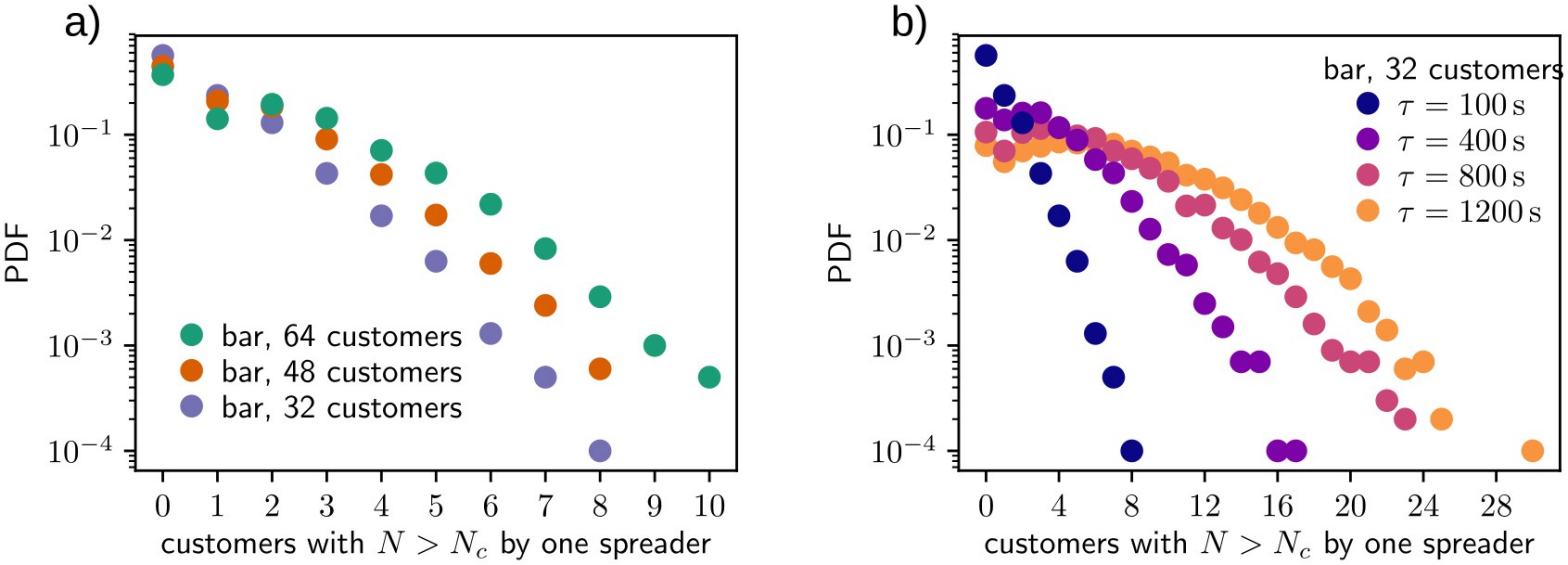
Distribution of the number of customers with critical exposure *N* > *N*_*c*_ caused by single spreader (a) from bar simulations with *τ* = 100 s and varying amount of customers and (b) from bar simulations with 32 customers and varying ventilation conditions via parameter *τ*.

One fundamental question in the spreading of the aerosols is how many critical spreading events originate from a single infected customer. To study this, Fig 4 shows the distribution of customers with *N* > *N*_*c*_ caused by a single spreader during one bar simulation. Now the figure shows the effect of more customer spots, as the average of caused critical exposure events is 0.74, 1.17 and 1.69 with 32, 48 and 64 customers in bar, respectively. Naturally this follows from the fact that, although the exposure distribution in Fig 3a stays the same, in the more crowded bar the customers sit more densely and more customers get affected by the spreader. The figure also gives an idea of what super-spreaders, i.e. the most efficient spreaders of the aerosols, can inflict: with the capacity of 64 customers, single spreader is observed to cause up to ten critical spreading events, while with the lowest capacity of 32 customers the number of critical events is still found to be as high as eight. This becomes quite evident in Fig 3b, where the slow-down of ventilation brings big changes in the tails of the customer numbers. Even though the increasing value of *τ* does not change the shape of the distributions it pushes the largest observed tail values almost linearly to higher and higher values.

To compare our results to Wells-Riley model (Eq 1), we obtain the ventilation rate *Q* by using the ACH associated to *τ* and calculating the system volume as the area (non-blocked, excluding shelves etc.) times some typical room height (3 m). Additionally, we estimate *q* from the aerosol generation rate of our infected customers: On average, the infected spread 5 aerosols/s ⋅ 3600 s/h + 6 ⋅ 40000 aerosols/h where the first term is from continuous emission and second from the discrete cough events. Then we convert this to estimate of *q* by dividing the sum with the critical exposure *N*_*c*_ thus leaving us with the number of ‘infecting’ quanta generated per hour by the infected, *q* = 25801/h. To compare Eq 1 to our simulations, we compute the infection probability as the proportion of customers with *N* > *N*_*c*_ from all customers that spent time *t* in the system simultaneously with the infected. This leads to a small error as the healthy customers can possibly accumulate exposure also after the infected has left the system because of lingering aerosols, but there is no reasonable way to take this into account. Thus we expect some level of underestimation from the Wells-Riley equation compared to the simulated risks. Finally, the pulmonary ventilation rate is approximated as $p\approx {\dot{V}}_{inh}$.

**Fig 5:**
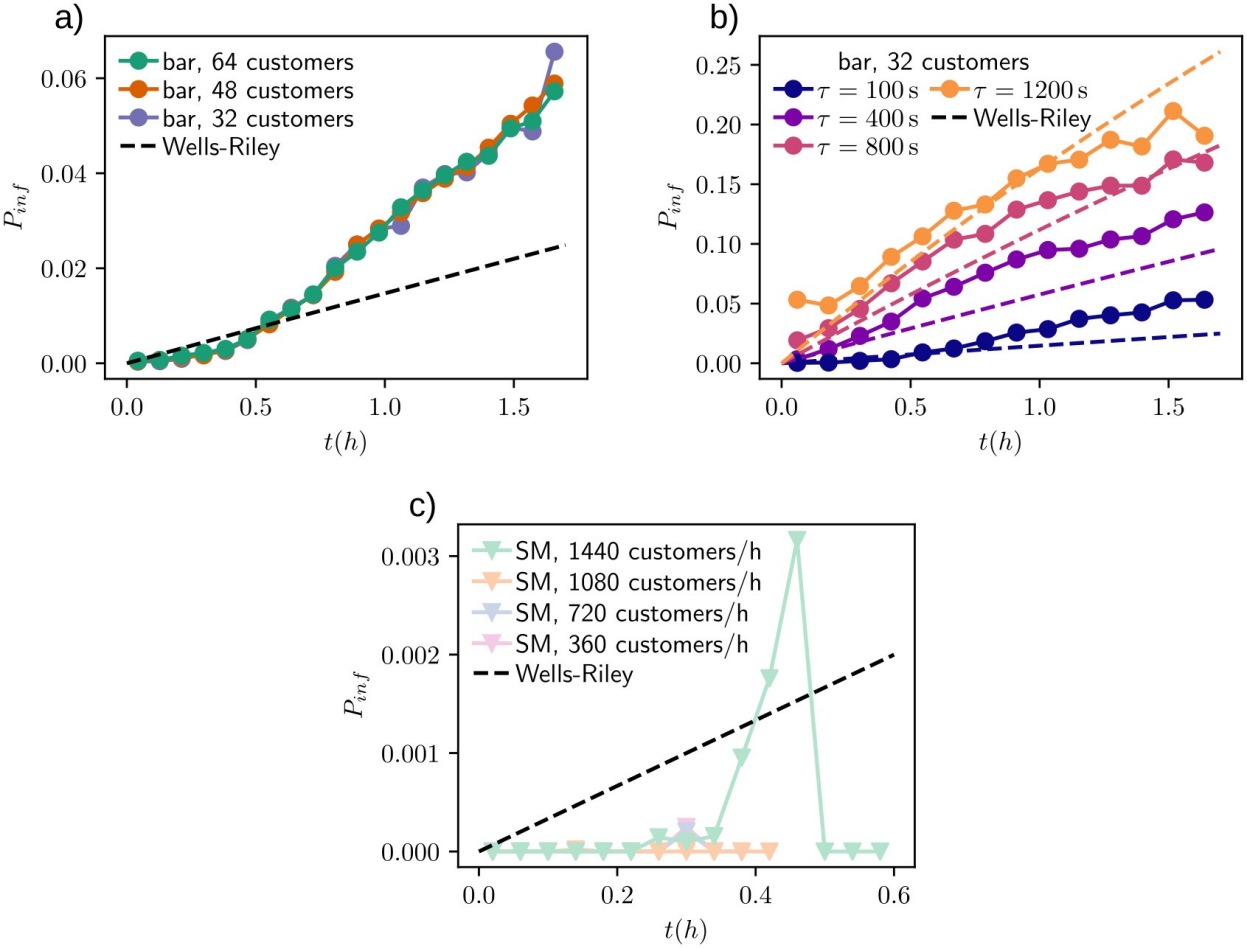
Approximate infection risk for healthy customers in (a) bar simulations with different customer densities, (b) bar simulations with varying ventilation (parameter *τ*) and (c) supermarket simulations with time *t* spent in the system with single infected customer. The infection probability in our simulations is estimated as the proportion of customers with *N* > *N*_*c*_. Comparison of simulation results to Wells-Riley equation is also included: the approximated value for quanta generation rate q = 25801/h is derived in the text, and the ventilation rate *Q* is calculated according to corresponding *τ* and Eq 3.

Fig 5 illustrates the infection probability for customers visiting the simulated systems as a function of time spent simultaneously with the infected customer and the Wells-Riley equation. In Fig 5a, the data is from bar simulations with varying number of customers and good ventilation *τ* = 100 s. Our simulated risk of critical exposure events follows Wells-Riley equation with small *t* but starts to overshoot after *t* > 0.5 h. As seen in Fig 5b, decreasing the ventilation rate in bar simulations by increasing *τ*, increases the infection risks significantly. The Wells-Riley equation, although not perfectly aligned with our results, follows the simulated risks quite closely, but in general we see that the mean-field like Wells-Riley underestimates easily the risk. This was partly expected due to the model details but is also due to the fact that the agents sample the environment in a way that enhances the risk. Thus, our model captures well the two common recommendations of risk avoidance: First, by shortening exposure times and spending less time in e.g. bar, the infection risks are lower. And second, better ventilation is crucial in lowering the risks. The SM simulations (Fig 5c) show no clear dependency between *t* and infection probability which is no surprise as critical spreading events in the simulations were highly unlikely. Notable here is also that the times customers spend in the SM simulations are much shorter than in the bar simulations.

Comparing our previous estimate of *q* to previous value obtained for COVID-19 [27], our estimate is significantly larger (a factor of ∼100). However, this arises from the model choices: In Wells-Riley model, *q* is a hypothetical measure of how many infectious doses are emitted by one infected person, and in our model we measure this by the aerosol amount scaled by the critical load *N*_*c*_. Here *N*_*c*_ is a rough estimate and as the eventual number of new infections and, therefore, the risk of getting infected are dependent on the used value of *N*_*c*_, our *q* is correspondingly only an approximation. To obtain a closer estimate of *N*_*c*_ would require extensive experiments that are out of scope for this work. In any case, the bar results validate the use of our estimate of *q*, as by fitting *q* from the data of Fig 5b we obtain *q*_*fit*_ = 26401/h which is almost same as our derived estimate.

**Fig 6:**
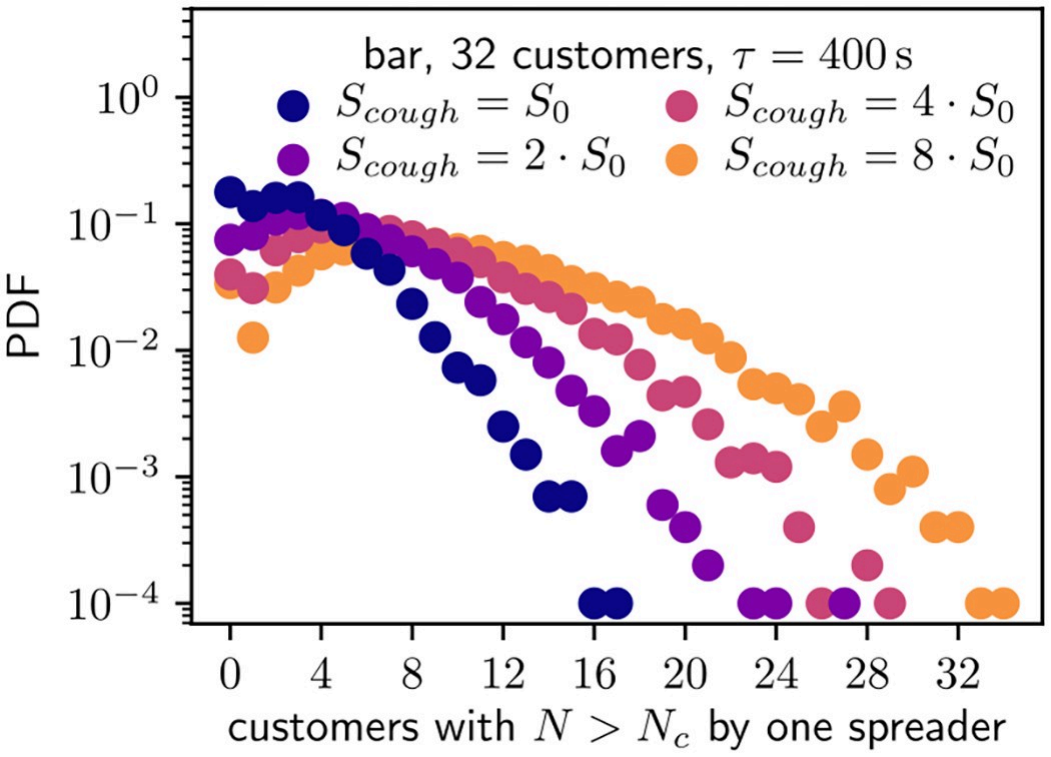
Distribution of the number of customers with critical exposure *N* > *N*_*c*_ caused by single spreader from bar simulations with 32 customers and *τ* = 400 s. Here the amount of emitted aerosols during a cough is varied from *S*_0_ = 40000 to 8 ⋅ 40000 aerosols.

As a final remark on the critical spreading events, we study how many critical exposure events originate from spreaders with increased aerosol output. This is closely connected to possible super-spreading events as person-dependent higher infecting rate is most likely linked to individual qualities such as aerosol emission during coughing and speaking. Therefore, we fix ventilation parameter *τ* = 400 s in bar with 32 customers and set the source term from coughing to multiples of control value *S*_0_ = 40000 aerosols. The distribution of critical events from single spreader with heightened emission of aerosols is presented in Fig 6. The figure shows that higher aerosol concentrations from coughs lead to same effect as slower room ventilation rate in Fig 5b: the distributions retain their shape but shift towards larger values. From the modeling perspective this is natural as both larger aerosol source terms and decreased ventilation lead to higher aerosol concentrations in the system.

### 3.3 Comparison to expected exposure from the zeroth order approximation

Finally, we compare our simulation results to the zeroth order, i.e. mean-field-like, approximation discussed in Section 2.4. For simulated cases we choose bar with 64 customers and SM with 1080 customers/hour with similar average customer densities. Comparing the average exposure from simulations to *N*_0_, we have

$$\begin{array}{cc} \frac{\left\langle N_{sim}^{bar}\right\rangle }{N_{0}^{bar}}\approx 6 & \;\frac{\left\langle N_{sim}^{SM}\right\rangle }{N_{0}^{SM}}\approx 8\cdot 10^{5}. \end{array}$$ (6)

**Fig 7:**
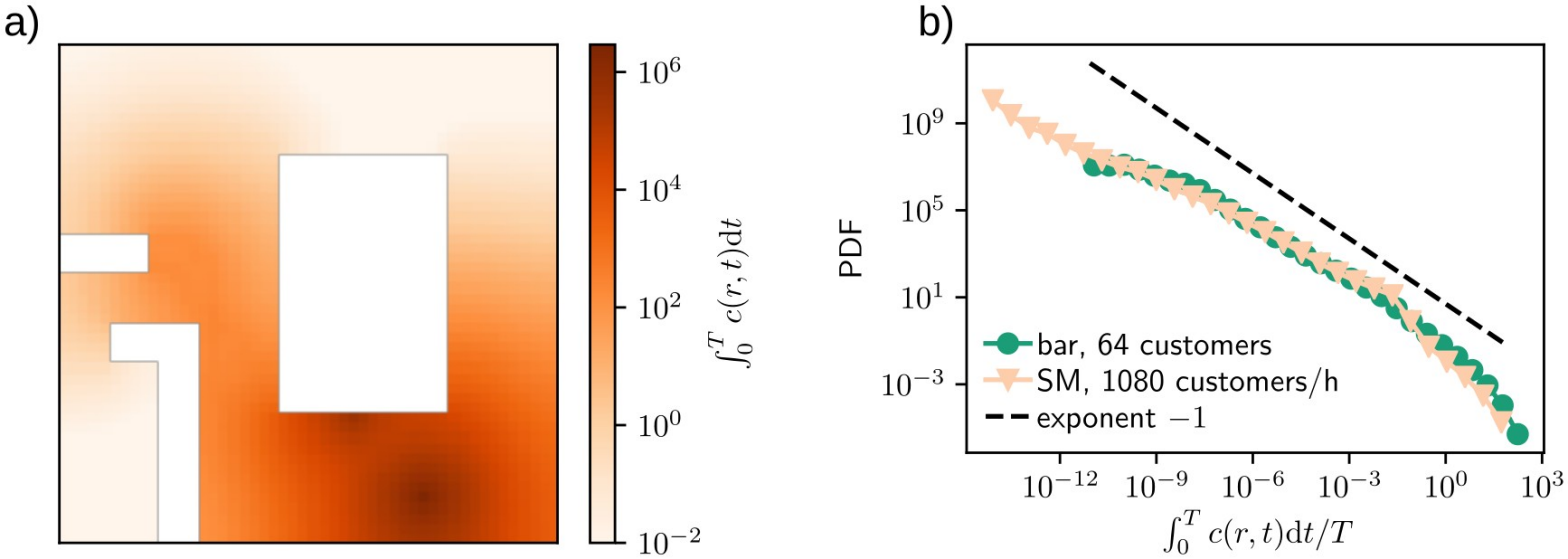
(a) The aerosol concentration summed through one single bar simulation. (b) The distribution of cell values seen in (a) divided by the corresponding simulation duration *T* collected from all of the simulations.

Next, we explore the background reasons for the discrepancy between the simulation and zeroth order approximation results. Instead of a steady-state field *c*(*r*, *t* → ∞), the healthy customers sample a concentration field that follows the spreader and highlights possible coughs and stops. For example, Fig 7a shows the integrated concentration field, $\int_{0}^{T}c\left(r,t\right)dt$ where *T* is the total simulation time, from a bar simulation with single spreader. The figure shows the seat of the spreader as the darkest area near bottom right corner but there are also notable concentrations near the bar counter and entrance. Combining the data from multiple simulations, Fig 7b illustrates the distribution of values of $\int_{0}^{T}c\left(r,t\right)dt/T$ for individual cells. In both bar and SM settings, the distributions are power-law-like spanning ∼13 (bar) to ∼15 (supermarket) decades. As the healthy customers accumulate their inhaled aerosols by sampling these distributions, they tend to visit cells that have been on the route of the spreader, e.g. the entrance and exit of bar in Fig 7a, which leads to larger exposures than the conservative zeroth order approximation. In SM this effect is even more dramatic, as the steady-state field *c*(*r*, *t* → ∞) at distance *r*_0_ is negligible. The distributions of the concentration fields also provide insight why the accumulated exposure distributions of Fig 3 are distinct in the two cases: both bar and SM have approximately same maxima of $P(\int_{0}^{T}c\left(r,t\right)dt/T)$ but in bar case the distribution is narrower than in SM leading to larger exposures.

## 4 Conclusions

Our goal has been to highlight the strength of agent-based simulations in grasping semi-quantitatively the risk levels of COVID-19 exposure in two typical settings. This of course also allows making observations with potentially practical interest. Due to the memory effects in our model and most likely also in real environments details prove to be important as Fig 7 demonstrates by the apparent similarity of the aerosol concentrations while the exposure measures are very different. It matters due to the non-linearity of the infection risk, which as such is also contained in the Wells-Riley model how *many times* and *for how long* a person samples these environments. The first effect is due to the high variation illustrated in the Figure, and the second one is inherent of infection models. In colloquial language, bar hopping is dangerous.

We thus obtain estimates of exposure and infection risk that are in line with expectations. Brief visits to supermarkets carry relatively low risk. Spending extensive amounts of time leads to an elevated risk for *some* but not all patrons due to how the “agents” sample the environment. This is also very visible in the comparison of the simulation results to the “expected” exposure. All in all our observations agree of course perfectly with the common recommendations of why and how to decrease extended close contacts with random people. They also point to the crucial role of ventilation in order to reduce aerosol concentrations, regardless of the environment [31, 32]. This is quite evident when *τ* is varied, and slow air circulation (ACH value small) leads to larger and larger risk levels. The largest “superspreading” events that one finds become thus also more substantial.
